# Caring for Rare Genetic Disease: A Vision for the Future

**DOI:** 10.1002/jgc4.70284

**Published:** 2026-09-15

**Authors:** Ruth Horn, Sarah L. Wynn, Sofia Douzgou Houge

**Affiliations:** ^1^ Ethics of Medicine, Faculty of Medicine Institute for Ethics and History of Health in Society, University of Augsburg Augsburg Germany; ^2^ Ethox Centre, Nuffield Department of Population Health University of Oxford Oxford UK; ^3^ Unique, Rare Chromosome Disorder Support Group Oxted UK; ^4^ Frambu unit Norwegian Centre for Rare Diseases Siggerud Norway; ^5^ Brain disorders unit Norwegian Centre for Rare Diseases Oslo Norway; ^6^ Department of Medical Genetics Haukeland University Hospital Bergen Norway

**Keywords:** advocacy, diagnosis, education, engagement, genomic medicine, healthcare, people living with a rare disease

## Abstract

The United Nations, 2021 resolution to promote and protect the human rights of the estimated 300 million People Living with a Rare Disease and their families, set a milestone worldwide. At the same time, the successful diagnostic results of large genomic initiatives are reshaping rare disease healthcare in many countries. However, increasing diagnostic capability does not necessarily translate into improved care. Patients, families, and healthcare professionals navigate challenges in variant interpretation and prognosis, uneven access to specialist expertise and follow‐up, limited natural history information and therapeutic options, and the wider familial and reproductive implications of genomic findings. Healthcare systems also face challenges related to workforce and service capacity, data governance, equity, research sustainability, and the integration of genomic technologies into longitudinal care. Drawing on published evidence, patient‐organization experience and illustrative clinical scenarios, we argue that the value of genomic medicine for rare disease should be assessed beyond diagnostic yield alone and across individual, clinical and societal levels. Our vision for the future includes accessible education for families and professionals, multidisciplinary and longitudinal rare disease services embedded within publicly funded healthcare, sustainable research and data‐sharing frameworks, partnership with patient advocacy organizations, and meaningful representation of people living with rare disease in governance, research, and innovation.

## Abstract (highlights)

**What Is Known About This Topic:**

The successful diagnostic results of large genomic initiatives with a focus on rare disease are shaping healthcare services in many countries. Clinical care for people living with rare genetic conditions and their families is practiced at the rapidly changing intersection between increasing use of genomic technologies, open access, web‐based resources and public sector participation in healthcare. However, the benefits of these initiatives in daily clinical practice for individual patients and their families, healthcare providers and systems are colored by both individual and systemic challenges.

**What This Paper Adds to the Topic:**

Data from patient organization Unique surveys show that receiving a diagnosis is very important for families, yet the day‐to‐day experience of having and caring for someone with a rare genetic disorder is described using mainly negative feelings. We argue that the benefits as well as the challenges of rare disease genomics in people's daily life and within society need to be considered at multiple levels. Our vision for the future includes an inclusive delineation of rare disease which considers not solely the (numerical) prevalence of the conditions but also the rare nuances that often accompany the experience of rare disease such as sociodemographic factors, language barriers, and technology literacy. This emphasis is important not only in the care for people with rare genetic conditions but also at a broader societal level to protect against possible harms and disadvantages such as genetic discrimination and allowing to build trust and outweigh harms and disadvantages. We also envisage educational programmes for both families and professionals, appropriate services as part of publicly funded healthcare, working in partnership with PAOs, and increased representation in governance structures and regulatory bodies that prepare programmes of change and innovation.

## 1 Introduction

On 16 December 2021, the United Nations adopted its first‐ever resolution recognizing the need to promote and protect the human rights of the estimated 300 million People Living with a Rare Disease (PLWRD) and their families worldwide (United Nations 2021; Nguengang Wakap et al. 2020); they face high levels of marginalization and reduced access to education, employment, and community participation. Families often experience fragmented care systems, limited coordinated support, and significant emotional and financial burdens (Ferreira 2019). This milestone was the outcome of coordinated and robust advocacy from rare disease civil society groups worldwide led by Rare Diseases International, the NGO Committee for Rare Diseases, and EURORDIS—Rare Diseases Europe.

Much effort is being channeled into large‐scale genomic diagnostic programmes to detect and diagnose rare diseases. A genetic diagnosis can substantially alter clinical management. A meta‐analysis reported clinical utility of genome sequencing of 77% (95% CI 64%–90%), while large‐scale data from the Deciphering Developmental Disorders (DDD) project showed management changes in 28% of children following a molecular diagnosis (Chung et al. 2023; Copeland et al. 2024). Successful diagnostic results from these approaches are increasingly informing medical genetics and rare disease services across Europe and beyond (Smedley et al. 2021; Wright et al. 2023; Laurie et al. 2025).

However, the benefits of these initiatives in daily clinical practice and everyday life for individual patients and their families, healthcare professionals, and healthcare systems are not always straightforward. Rare diseases are often complex and multisystemic, making diagnosis, long‐term management, and care coordination particularly challenging (Ferreira 2019; Faye et al. 2024). Clinical care is increasingly delivered at the intersection of genome‐wide testing technologies, rapidly expanding web‐based information resources, social media, generative artificial intelligence (AI), and publicly funded healthcare systems (Laurie et al. 2025; Emmert‐Streib 2024). Within this evolving landscape, access to timely diagnosis, specialist expertise, and appropriate follow‐up may depend on local resources and individual circumstances rather than on equitable, evidence‐based care pathways. At the same time, core elements of high‐quality genetic care—including genetic counseling, longitudinal support, multidisciplinary collaboration, and patient‐centered communication—risk being undervalued despite their importance to patients and families (Clarke and Harper 2020; Andersen et al. 2025; Merker et al. 2021). The absence of established care pathways for PLWRD may also limit opportunities to educate and equip healthcare professionals with the knowledge and skills required to provide high‐quality rare disease care (Andersen et al. 2025).

In this Perspective, we argue that the value of genomic medicine in rare disease should be considered beyond diagnostic success alone. We examine the opportunities and challenges of contemporary genomic medicine from the perspectives of patients and families, healthcare professionals, and healthcare systems. We first consider the experience and implications of receiving a rare genetic diagnosis, including communication of results, uncertainty, expectations, and consequences for the wider family. We then discuss challenges related to longitudinal clinical care, healthcare resources, equity, data governance, and research. Finally, we outline a vision for the future of rare disease care, emphasizing education, multidisciplinary and longitudinal care, partnership with Patient Advocacy Organizations (PAOs), equitable access to services, and meaningful involvement of PLWRD in shaping policy, research, and innovation.

## 2 Current Practice of Genomic Medicine for Rare Disease

National initiatives across Europe and the United Kingdom—including the NHS England Genomic Medicine Service and the 100,000 Genomes Project, the French Genomic Medicine 2025 plan, and programmes in Germany and Denmark—have embedded or piloted large‐scale sequencing to improve rare disease diagnosis (Horn et al. 2023; Smetana and Broz 2022). Carrier screening programmes are also expanding from approaches targeted by family history or population prevalence toward broader population screening (Dive et al. 2022). Genomic programmes that are disease‐ or gene‐agnostic are promising for this cohort of patients, and accounts of positive outcomes are widely shared and promoted.

Yet alongside the potential advantages of genomic approaches, implementation also brings challenges, and benefits are not always straightforward in daily practice for patients and families, healthcare professionals, healthcare systems, or wider society (Horn et al. 2025). Diagnostic performance depends not only on the sequencing assay but also on the quality of phenotypic information, availability of relatives, and the analytical strategy. In the DDD project, diagnostic rates were lower among probands not recruited in a family trio, families with multiple affected members, probands of non‐European ancestry, and those with high consanguinity; composite and partial diagnoses also suggested that more than one genetic or nongenetic factor may contribute to the phenotype (Wright et al. 2023).

Whole‐genome sequencing can broaden the range of detectable variation, but it shifts rather than removes the interpretive burden. In the 100,000 Genomes Project pilot, 14% of diagnoses required a combination of research and automated approaches, including cases involving non‐coding, structural and mitochondrial variants or coding variants poorly covered by exome sequencing (Smedley et al. 2021). The diagnostic question is also timing dependent. Systematic reanalysis of the pan‐European Solve‐RD resource produced new diagnoses because disease genes had been newly reported, ClinVar classifications had changed, expert review supported reclassification, or bespoke bioinformatic analyses identified previously missed variant types (Laurie et al. 2025). More advanced approaches may therefore improve yield while simultaneously increasing the need for specialist bioinformatics, functional interpretation, multidisciplinary expertise, and international data sharing (Yepez et al. 2025).

At service level, bottlenecks can occur before, during, and after sequencing. Early implementation of the NHS Genomic Medicine Service identified burdensome consent and test‐ordering processes, delays while obtaining parental samples, administrative backlogs, long turnaround times, shortages of clinical scientists and genetic counselors, and poor interoperability between genomic information systems and electronic patient records (Friedrich et al. 2024). Mainstreaming genomics also requires nongenetics professionals to have sufficient confidence and training to select tests, explain their implications, and act on results (Friedrich et al. 2024). Scaling sequencing without parallel investment in clinical genetics, counseling, laboratory and bioinformatics capacity, digital infrastructure, and post‐test follow‐up therefore risks moving the bottleneck from generating genomic data to interpreting, communicating, and acting on it (Horn et al. 2025; Friedrich et al. 2024; Goranitis et al. 2025).

## 3 Experiencing a Rare Genetic Diagnosis

Many patients and their families who receive a genetic diagnosis report benefits that extend beyond direct changes in medical management. These may include treatment or surveillance implications, access to appropriate services and support groups, connection with others living with the same condition, greater understanding and validation of their experiences, reproductive decision‐making, future planning, and relief from uncertainty or feelings of guilt (Copeland et al. 2024; Modelhart et al. 2024; Lemke et al. 2026; Genetic Alliance UK 2022).

These benefits are neither uniform nor easily captured by a single concept of utility. Clinical utility and personal utility overlap but are not interchangeable: a diagnosis may influence clinical management without necessarily being experienced as personally useful, while a diagnosis with little immediate effect on treatment may nevertheless be highly valued because it provides explanation, facilitates planning or enables social connection (Lemke et al. 2026; Bunnik et al. 2015; Watts and Newson 2026; Bowman‐Smart and Carter 2026). Conversely, receiving a diagnosis may introduce new uncertainties or concerns, and information may not always support meaningful decisions or action in the circumstances of an individual patient or family (Bunnik et al. 2015; Watts and Newson 2026).

The value of diagnosis therefore depends not only on obtaining a laboratory result, but also on how that result is communicated and supported. Genetic diagnosis may be better understood as a process rather than a single disclosure event, encompassing explanation of the result and its uncertainties, opportunities for patients and families to ask questions and revisit its implications over time, and appropriate clinical, psychosocial and practical support. Studies of families living with rare genetic conditions emphasize the importance of timely, understandable and individualized information, empathetic and respectful communication, psychological support, and a trusting relationship with healthcare professionals (Merker et al. 2021; Modelhart et al. 2024; Burbury et al. 2025). Written information and links to reliable sources, specialist services and relevant patient communities may also help families understand and integrate a diagnosis into everyday life. When such information, continuity and support are lacking, patients and families may continue to experience uncertainty and isolation even after the diagnostic odyssey has formally ended (Modelhart et al. 2024; Genetic Alliance UK 2022).

Accordingly, post‐test support should include timely discussion of the result, sensitive and understandable language, opportunities for follow‐up, and sources of further information and psychosocial support (Merker et al. 2021; Genetic Alliance UK 2022; Burbury et al. 2025). When these are lacking, some patients and families report continuing isolation and uncertainty after diagnosis (Modelhart et al. 2024; Unique – Rare Chromosome and Gene Disorder Support Group 2025). Case example 1 illustrates how obtaining a molecular diagnosis does not necessarily translate into timely access to specialist information, counseling, and practical support.

For many newly recognized rare diseases, published clinical observations remain limited and are often derived from small cohorts assembled across different healthcare systems. Longitudinal natural history data—particularly regarding adulthood and later‐life manifestations—as well as systematically collected patient‐ and family‐reported outcomes are frequently lacking (Adang et al. 2024). The rarity of individual conditions also limits exposure and disease‐specific expertise within individual healthcare institutions, even those serving large populations, making national and international collaboration and concentration of expertise particularly important (Faye et al. 2024; Adang et al. 2024).

## 4 Addressing Expectations

Genome sequencing does not provide a clear diagnosis for all people with suspected rare genetic disease (Smedley et al. 2021). It is vital to continue research to improve diagnostic yield and the speed at which patients access genomic testing, while also supporting those who remain undiagnosed. Advances in genomics can generate renewed hope among families who do not receive a diagnosis, including hope that one may become possible in the future (Gurasashvili et al. 2024). The discovery that *de novo* pathogenic variants in a critical region of the non‐coding gene *RNU4‐2* explain around 0.4% of neurodevelopmental disorders now known as ReNU syndrome illustrates the value of continued analysis (Chen, Dawes, et al. 2024). While hope can be an important coping resource, it is also important to manage expectations and avoid overly positive rhetoric that may create unrealistic expectations (Wiles et al. 2008).

**FIGURE 1:**
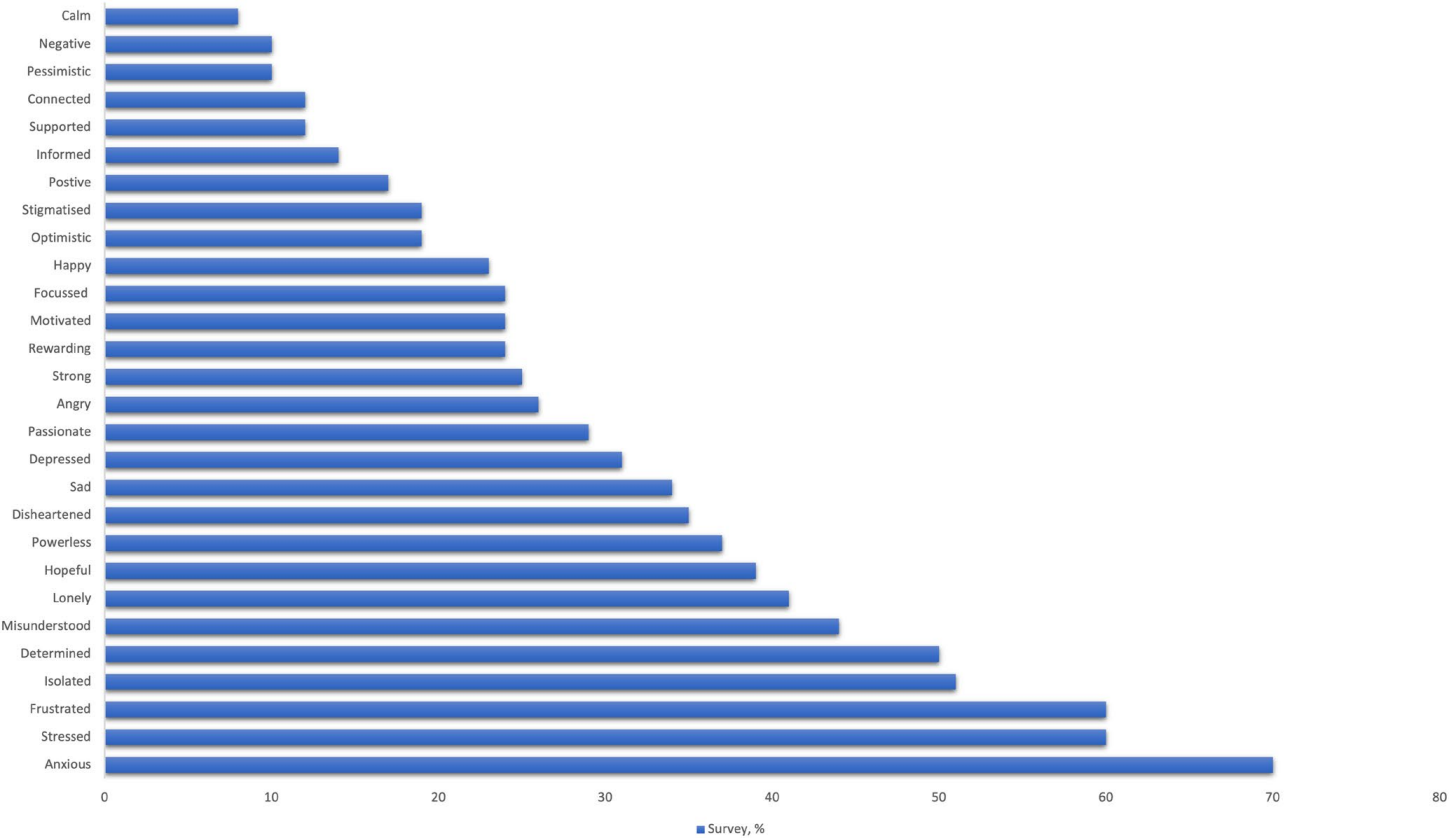
Unique survey: From the list of words below, which best describe your experiences of having or caring for someone with a rare chromosome or gene disorder. 2154 responses; 61% UK, 15% USA, 5% Australia, 2.5% Canada, 1.6% Ireland, 1.6% New Zealand (56 countries in total).

Furthermore, receiving a diagnosis does not end concerns for all patients and families. Some report frustration when a diagnosis is followed by limited prognostic information, no clear clinical care pathway, or no available treatment, clinical trial, or research opportunity (Andersen et al. 2025; Rosell et al. 2016). Such experiences may be associated with anxiety, frustration, isolation, and loss of hope (Rosell et al. 2016). Survey data from Unique, a UK‐based charity for people and families living with rare chromosome and gene disorders, show a high frequency of negative feelings: 70% reported anxiety, 60% frustration, 60% stress, and 51% isolation (Figure 1) (Unique – Rare Chromosome and Gene Disorder Support Group 2025).

The way information is provided before and after testing about possible benefits and limitations is therefore important for managing expectations and preparing patients and families for uncertainty, further investigations and potentially limited prognostic information (Zybarth et al. 2024). People should also be prepared for the possibility that a rare genetic diagnosis may not change immediate clinical management and should receive appropriate counseling about potential reproductive implications and available options, where relevant (Clarke and Harper 2020; Dive et al. 2022; Silver and Norton 2021). The way this information is perceived by families is highly variable. Case example 2 illustrates how perceptions of disease severity, lived experience and reproductive preferences may differ between families and healthcare professionals, and how access to reproductive technologies may vary between services.

For many families, one important expectation following diagnosis is the possibility of a targeted treatment (Andersen et al. 2025; Unique – Rare Chromosome and Gene Disorder Support Group 2025). Clinical trials are increasingly being undertaken in rare genetic disorders and are essential for assessing the efficacy and safety of experimental therapies (Hipp et al. 2025). Rare disease trials nevertheless face distinctive challenges, including small and geographically dispersed populations, limited natural history data and validated endpoints, international coordination, travel burden, funding, and health‐system capacity to support trial‐related care (Kempf et al. 2018; Medicines and Healthcare products Regulatory Agency 2025). Case example 3 illustrates how access to emerging therapies for ultrarare disorders may depend not only on scientific evidence or clinical eligibility but also on institutional capacity, funding, coordination across services, and the sustainability of research programmes.

Public and patient education has become an increasingly visible component of genomic and precision medicine. The European Society of Human Genetics Education Committee provides examples of professional society‐led public engagement and educational activities (Tobias et al. 2021). Social media and condition‐specific websites are important channels for information exchange within many PAOs, which increasingly contribute to education, research, and partnership activities (Patterson et al. 2023). Rare disease education, engagement, and support are also explicit functions of national or regional rare disease centers, including the Norwegian National Centre for Rare Diagnoses and the Manchester Rare Conditions Centre in the United Kingdom (Nasjonalt senter for sjeldne diagnoser 2026).

## 5 Managing Genetic Findings

Once genomic testing results are available, clinicians may face difficult questions about the significance of reported findings. Variant interpretation entails understanding mechanisms such as variable expressivity, incomplete penetrance, mosaicism, epigenetic effects, environmental influences, and modifier or additional genetic factors. For example, different molecular subtypes of Noonan syndrome are associated with distinct patterns and severity of clinical manifestations (Tartaglia et al. 2002). In Treacher Collins syndrome, individuals carrying pathogenic TCOF1 variants may occasionally have extremely mild or apparently absent clinical manifestations (Teber et al. 2004). In SMAD6‐related craniosynostosis, at least 20 of 26 rare damaging variants in one cohort were inherited from an unaffected parent, illustrating marked incomplete penetrance; a previously proposed common modifier did not predict phenotype in this independent cohort, underscoring the need to validate modifier effects before clinical use (Calpena et al. 2020).

Further examples illustrate distinct mechanisms of phenotypic complexity. In PIK3CA‐related overgrowth spectrum, postzygotic mosaicism contributes to clinical heterogeneity: the developmental timing of the pathogenic event and the tissues involved influence the phenotype, and low‐level variants may require deep sequencing of affected tissue rather than blood (Douzgou et al. 2022). In TET3 deficiency, a genome‐wide DNA hypermethylation episignature distinguished affected from unaffected individuals and provided functional evidence useful for classifying uncertain genotypes, showing how epigenomic data can refine variant interpretation (Levy et al. 2021). Environmental exposures may create additional phenotypic overlap; among children referred with suspected fetal alcohol spectrum disorders, chromosome disorders are an important alternative diagnosis, illustrating why exposure history, phenotype, and genomic findings need to be considered together (Douzgou et al. 2012).

Genetic testing can also remain inconclusive because of technical limitations, incomplete understanding of variant pathogenicity and disease mechanisms, and limitations in interpreting non‐coding and structural variation (Wojcik et al. 2023). Variants are designated variants of uncertain significance (VUS) when evidence is insufficient to classify them as pathogenic or benign; importantly, a VUS should not be used to guide clinical decision‐making (Richards et al. 2015). Uncertain genomic results can cause confusion for PLWRD and families, particularly when people approach genomic testing with more deterministic than probabilistic expectations (Raz et al. 2022).

A further challenge for both clinicians and patients is the limited natural history information available for many rare and ultrarare diseases. Patient advocacy organizations increasingly contribute to research readiness through patient registries, natural history initiatives and partnerships with researchers and industry (Patterson et al. 2023). Robust natural history data can reduce uncertainty in therapeutic development by characterizing disease progression, identifying clinically meaningful outcomes and biomarkers, and informing trial design (Kempf et al. 2018; Liu et al. 2022).

## 6 Considering the Context of a Rare Genetic Finding

The diagnosis of an inherited rare disease may be clinically relevant not only for the patient but also for biological relatives. Some pathogenic or susceptibility copy‐number variants identified in children are inherited from a parent, creating implications for the parent and wider family (Burghel et al. 2020). Legal and professional expectations regarding communication of familial genetic risk differ across jurisdictions. Comparative work describes important differences between France, Germany, and England in how duties to inform relatives and confidentiality are framed (Horn et al. 2023). Regardless of jurisdiction, professionals may want to prepare patients for the possible wider impact and discuss the potential implications of a genetic finding for relatives. This can place a “weight of responsibility” on families regarding whether and when to disclose information. Early discussion can support patients in considering which relatives may benefit from information and how this might be communicated (Royal College of Physicians, Royal College of Pathologists, British Society for Genetic Medicine 2019). The availability of parental samples has also contributed substantially to diagnostic interpretation in rare disease genome sequencing (Wright et al. 2023). However, trio‐based approaches can create practical and equity challenges in specific social contexts, including single‐parent families, adopted children, and children conceived using unknown gamete donors.

The challenge of returning information may be increased when genetic testing for a rare disease occurs in a hybrid clinical‐research context (Hallowell 2018). Such settings challenge traditional consent models and blur distinctions between clinical care and research. Consent models and regulatory approaches are evolving in response to these difficulties (Gaille et al. 2021). The UK 100,000 Genomes Project provides an important example of these clinical‐research hybrid practices and the governance challenges they create (Dheensa et al. 2018).

## 7 Prioritizing Healthcare Resources

While large‐scale genomic programmes focus on improving diagnosis and treatment of rare diseases, competition for healthcare resources remains a challenge even in historically solidaristic national healthcare systems such as those in the United Kingdom and Scandinavia (Brean 2024). Telehealth solutions are increasingly adopted as substitutes or complements to face‐to‐face healthcare. However, concerns remain that they may amplify inequities related to access to technology, digital literacy, geography, language, and other social determinants of health (Chen, Hartman, et al. 2024).

Studies evaluating the impact of genomic sequencing on overall healthcare expenditure remain limited. Although sequencing may streamline diagnostic pathways, enable prevention and support targeted treatment, evaluation of genomic medicine also needs to account for infrastructure, consent, interpretation, follow‐up, long‐term surveillance and the management of additional findings. Assessing these costs alongside clinical and personal benefits remains a challenge for health economists, policymakers, clinicians and PLWRD (Goranitis et al. 2025). There are also concerns that investment in genomic screening can compete with other healthcare and social priorities, particularly in resource‐constrained systems (Horn et al. 2025).

## 8 Safeguarding Rare Genetic Data

Fears about disadvantage, discrimination and loss of privacy can undermine public trust and willingness to participate in genetic screening, genomic sequencing and data sharing. Genomic data can carry re‐identification risks, and technical safeguards alone cannot guarantee absolute anonymity (Wan et al. 2022). Because rare disease research often depends on national and international data sharing, governance needs to regulate who can access genomic data and for what purposes, while providing meaningful safeguards against misuse and discrimination.

To build trust in genetic and genomic initiatives, it is also important that PLWRD can benefit equitably from them. With an estimated 300 million people living with a rare disease worldwide, the collective burden is substantial (Nguengang Wakap et al. 2020). Rare disease stigma remains an international public health concern (Pearce and Baynam 2024). Genomic research datasets remain disproportionately derived from populations of European ancestry and people with greater access to specialist services, contributing to inequities in variant interpretation and genomic medicine (Serrano et al. 2023). One example is cystic fibrosis, where epidemiological data, registries, and newborn screening remain less complete in parts of North Africa and other underrepresented regions, contributing to diagnostic and knowledge gaps (El Makhzen et al. 2023).

## 9 Fostering Rare Research and Development

Rare‐disease research is supported through funding mechanisms operating at very different scales. NORD provides competitive international seed grants, often disease‐specific and funded largely by PAOs, while the Foundation for Rare Diseases in France launches several calls each year and supports both research projects and access to specialized technology platforms (National Organization for Rare Disorders 2026). At a different scale, ERDERA is a Horizon Europe partnership with an estimated budget of EUR 380 million through 2031 (European Commission 2024a). Such schemes provide important opportunities, but their scale, consortium requirements and application structures may create barriers for smaller research groups and disease communities with limited grant‐development capacity (European Commission 2024a). Funding gaps therefore remain particularly relevant for early‐stage and disease‐specific research, where patient organizations and small grants may provide critical seed support (Patterson et al. 2023; National Organization for Rare Disorders 2026).

Patient advocacy organizations for rare genetic diagnoses, exceptionally but increasingly, self‐organize in foundations with global reach, funding innovative research. Following the discovery of ReNU syndrome, ReNU Syndrome United was established as an international patient organization supporting community, education, research and engagement initiatives (Andersen et al. 2025; ReNU Syndrome United 2026). Importantly, the great majority of rare disease PAOs do not have their own research funds and heavily rely on donations, fundraising, and voluntary work (Patterson et al. 2023).

## 10 A Vision for the Future

Based on the challenges described above, our wish list for the future includes building an inclusive delineation of rare disease which considers not solely the numerical prevalence of conditions but also the rare nuances that often accompany the experience of rare disease, such as sociodemographic factors, language barriers and technology literacy. Specific efforts are needed to design and deliver medical education on rare disease to address the unmet needs of both early‐career and experienced healthcare professionals and to build a “rare workforce”. We need public healthcare approaches that quantify value as well as cost, and we envisage protected or dedicated rare disease services within public healthcare, supported by the framework of the European Reference Networks. The JARDIN Joint Action, launched in 2024 for the 2024–2027 period, is specifically intended to support integration of European Reference Networks into national healthcare systems and improve the sustainability and accessibility of specialized rare disease care (European Commission 2024b). We strongly believe that healthcare professionals and academics should work in partnership with rare disease PAOs to collate their knowledge and experience and to influence governments and funding bodies. The rare disease community should also be meaningfully represented within governance and regulatory structures that shape programmes of change and innovation: “nothing about us without us.”

A number of aspects need to be taken into account to address possible negative effects: unrealistic expectations should be carefully managed; the broader implications of genetic/genomic testing for rare disease including subsequent options should be communicated prior to testing and when returning information; economic cost–benefit analyses need to be carried out including the perspectives of individual patients as well as the broader society; social security safety nets should be guaranteed to avoid discrimination and stigmatization; and a renewed focus on the issue of equity and inclusion of deprived or marginalized populations is needed. Importantly, we strongly believe that all approaches should include both a patients‐and‐families based experience and educational and training opportunities for developing professionals.

## 11 Conclusion

In conclusion, we see that the benefits as well as disadvantages or potential harms of rare disease genetics and genomics in people's daily life and within society need to be considered at various levels. The successful development and implementation of genetic/genomic screening programs aiming to improve diagnosis, treatment, and overall quality of life with rare disease requires the positive effects to outweigh the negative effects, and patients must be able to perceive the benefits to build trust.

This means not only more research studies exploring the advantages and disadvantages at various levels but also choices at the political and policy‐making level must take these studies into account to address and counterbalance any negative effects.

## 12 Illustrative Case Examples

The following examples are illustrative scenarios informed by mixed sources, including clinical experience and situations encountered or reported in rare disease care. They do not represent individual identifiable patients or families and are not presented as research data. Details have been combined or modified where appropriate. Their purpose is to illustrate how diagnostic, reproductive, organizational, and healthcare‐system factors can interact in the care of people living with rare genetic conditions and their families.

### 12.1 Case Example 1: Diagnostic Information Without Timely Specialist Support

A couple who lives abroad receives the diagnosis of a very rare (prevalence 1–9/100000), neurodevelopmental genetic syndrome in their firstborn with moderate, global developmental delay by their pediatrician who requested the test. They have many questions yet they are told they have to wait 4 months to speak with «someone who knows more about the condition» – there is a long queue for post‐test genetic counseling appointments and they are not prioritized as urgent. Waiting for the appointment, they look up available web‐based resources that do not match the symptoms of their child and list a number of symptoms they are unsure their child has or may develop. They are struggling to manage the sleep disorder their child manifests in the meantime. The child has a neurodevelopmental assessment and receives an additional diagnostic code of «autism». They get unexpectedly pregnant. They arrive at the planned genetics appointment asking for an urgent referral for termination of pregnancy.

### 12.2 Case Example 2: Reproductive Choice, Perceived Disease Severity and Variation Between Services

The newborn, first child of a Northwest European couple is referred because of the diagnosis of a very rare genetic cause of isolated albinism.

Albinism is a heterogeneous group of nonprogressive genetic disorders characterized by reduced or absent ocular pigmentation and variable skin pigmentation. In more complete forms, hair, eyelashes, and eyebrows may be very lightly pigmented and the skin may tan poorly. Reduced melanin increases susceptibility to ultraviolet radiation and skin damage, making lifelong sun protection important (Marcon and Maia 2019). Syndromic forms of albinism also occur and may require additional clinical surveillance; genetic testing can help distinguish these from non‐syndromic forms (Marcon and Maia 2019). People with albinism may experience stigma, prejudice, and discrimination related to visible difference, despite substantial advocacy efforts (Pearce and Baynam 2024; Marcon and Maia 2019).

The couple comes to the appointment without the child and requests to be referred for assisted reproduction using preimplantation genetic diagnosis for albinism because they are convinced that their child and the family will experience significant stigma and adverse educational and life effects. Their request is discussed at the local clinical meeting and is declined: the first local multidisciplinary team concludes that the relevant eligibility or severity criteria are not met to confer indication for pre‐implantation or prenatal genetic diagnosis. The family takes their case to a different city/genetic service and their request is accepted; another service reaches a different decision.

### 12.3 Case Example 3: Access to Experimental Treatment and the Burden of Care Coordination

The parents of a newborn with in vitro fertilization are referred to genetics because of a diagnosis of an ultrarare genetic syndrome with very poor prognosis. Almost simultaneously, an experimental treatment is published with positive developmental results in older patients. The parents decline the genetics appointment saying they have many different clinical appointments to attend already. Shortly afterwards they get unexpectedly pregnant naturally and ask for a re‐referral to genetics. They opt for prenatal diagnosis of the new pregnancy and ask if the experimental treatment could be made available for their child. The lead physician contacts the hospital's clinical research trial unit and is told there is no capacity. The international clinical trial lead abroad is willing to include the child if the family's travel is funded and some clinical protocol investigations can be taken forward by the local hospital. The lead physician writes to the hospital director for financial support, attends the hospital's ethics committee meetings, organizes the local follow‐up protocol and the group of colleagues who will be taking this forward and explains the process to the family who consent to experimental treatment and can travel. One year into treatment, the pharmaceutical company announces a change in internal policy which stops the clinical trial and the child's treatment transitions to a compassionate use program to be taken forward locally and on top of the regular clinics of all physicians involved.

## Author Contributions

**Sofia Douzgou Houge:** conceptualization, methodology, funding acquisition, writing – original draft, writing – review and editing, project administration. **Sarah L. Wynn:** resources, writing – review and editing, writing – original draft, investigation, data curation. **Ruth Horn:** funding acquisition, writing – original draft, methodology, writing – review and editing.

## Funding

This work was supported by Norges Forskningsråd, 358387. Norwegian National Centre for Rare Diseases, 43066. Economic and Social Research Council, ES/T00908X/1. Deutsche Forschungsgemeinschaft, 529272624.

## Disclosure

No generative AI or AI‐assisted technologies were used in the preparation of this manuscript.

## Ethics Statement

This study was performed according to the Declaration of Helsinki and approved by the Western Norway Regional Ethics Committee (REC 604007).

## Consent

This study utilized data from anonymous surveys, ensuring that no personally identifiable information was collected. Participants were informed of the study's purpose and that completion of the survey constituted voluntary informed consent. No further written patient consent was required for this study as it did not involve human participants, individual patient data, or confidential samples.

## Conflicts of Interest

The authors declare no conflicts of interest.

## Data Availability

The data that support the findings of this study are available on request from the corresponding author. The data are not publicly available due to privacy or ethical restrictions.
